# Endoscopic Diagnosis of Primary Intestinal Lymphangiectasia in an Adult With Refractory Protein-Losing Enteropathy: A Case Report

**DOI:** 10.7759/cureus.66141

**Published:** 2024-08-04

**Authors:** Zeyad Khalil, Hamza N Ali, Hosam Ibrahim, Eman Z Al-Abbedien

**Affiliations:** 1 College of Medicine, October 6 University, Cairo, EGY; 2 Geriatrics, Beni Suef Hospital, Beni Suef, EGY

**Keywords:** medium-chain triglycerides, refractory edema, endoscopy duodenal biopsy, nutritional therapy, gastrointestinal disorder, protein-losing enteropathy, primary intestinal lymphangiectasia

## Abstract

Primary intestinal lymphangiectasia (PIL) is a rare disorder characterized by dilated lymphatic vessels in the gastrointestinal tract, leading to protein-losing enteropathy (PLE). We report the case of a 43-year-old male presenting with refractory PLE, characterized by generalized edema, chronic diarrhea, and significant weight loss over four months. Despite multiple consultations and treatments for presumed inflammatory bowel disease, his symptoms persisted, and his condition worsened. An upper endoscopy was performed, revealing white villi in the duodenum. Histopathological examination of duodenal biopsies confirmed the presence of markedly dilated lymphatic vessels in the lamina propria, consistent with PIL. The patient was managed with a high-protein, low-fat diet supplemented with medium-chain triglycerides (MCTs) and octreotide therapy. This treatment regimen led to significant clinical improvement, including reduced edema, normalization of albumin levels, and resolution of gastrointestinal symptoms. This case underscores the importance of considering PIL in adults with refractory PLE.

## Introduction

Primary intestinal lymphangiectasia (PIL) is a rare, chronic condition characterized by dilated lymphatic vessels in the intestinal mucosa, leading to excessive loss of lymphatic fluid into the gastrointestinal lumen, subsequently causing protein-losing enteropathy (PLE). First described by Waldmann in 1961, PIL primarily affects children, although adult-onset cases are increasingly being recognized [1]. PIL with PLE is exceptionally rare in adults. While this condition is more commonly reported in pediatric populations, adult cases are infrequent, with fewer than 100 documented instances in the literature [2]. The rarity can be attributed to several factors; adults often present with non-specific symptoms such as abdominal pain, diarrhea, and weight loss, which overlap with more common gastrointestinal disorders, leading to delays in accurate diagnosis. Moreover, the condition may be misinterpreted due to its subtle endoscopic findings, contributing to diagnostic challenges. While congenital PIL is rare, acquired forms associated with malignancies, infections, or post-surgical complications, although uncommon, are distinct from the primary idiopathic PIL seen in adults. PIL results from an abnormality in the lymphatic system of the gastrointestinal tract. The lymphatic vessels, which normally function to transport lymph, a fluid rich in proteins, lipids, and immune cells, become dilated and dysfunctional. This dilation causes lymph to leak into the intestinal lumen instead of being transported properly to the thoracic duct and back into the bloodstream [2]. The leakage of lymphatic fluid into the gut lumen leads to a significant loss of plasma proteins, particularly albumin, immunoglobulins, and lymphocytes, resulting in hypoproteinemia, hypoalbuminemia, and lymphopenia [1]. These biochemical abnormalities manifest clinically as generalized edema, ascites, and chronic diarrhea [3].

The pathophysiological mechanism involves a combination of mechanical and inflammatory factors. Mechanically, the increased pressure in the lymphatic system causes dilation and rupture of lymphatic vessels. Inflammatory cytokines and other mediators released during the process further exacerbate the leakage of lymphatic fluid [3]. On a biochemical level, the loss of albumin and other plasma proteins reduces the oncotic pressure in the blood vessels, leading to fluid extravasation into the interstitial spaces, which is clinically observed as edema [4]. The loss of immunoglobulins and lymphocytes compromises the immune system, making patients more susceptible to infections [5]. The dietary fat absorbed by the intestines is also lost through the lymph, leading to malabsorption and steatorrhea [5].

Endoscopy often reveals characteristic white villi due to dilated lacteals, and histopathological examination confirms the diagnosis by showing markedly dilated lymphatic vessels in the lamina propria [6]. The standard treatment for PIL involves dietary modifications, including a high-protein, low-fat diet supplemented with medium-chain triglycerides (MCTs), which are absorbed directly into the portal circulation bypassing the lymphatics. Pharmacological treatments such as octreotide, a somatostatin analog, have shown efficacy in reducing lymphatic leakage and improving clinical symptoms [7]. This case underscores the importance of considering PIL in the differential diagnosis of adult-onset PLE and highlights the role of endoscopy with targeted biopsies in establishing a definitive diagnosis.

## Case presentation

A 43-year-old male presented to the gastroenterology clinic with complaints of generalized edema, chronic diarrhea, and significant weight loss over four months. He reported progressive swelling in his legs and abdomen, along with persistent, non-bloody diarrhea that occurred multiple times daily. The patient had lost approximately 10 kg during this period. He initially visited his primary care physician twice over the past three months and was treated symptomatically for presumed inflammatory bowel disease with no improvement.

The patient had no significant past medical history and no known allergies. He was a non-smoker and did not consume alcohol or use recreational drugs. He worked as an accountant and had no known exposure to environmental toxins. His family history was unremarkable, with no history of gastrointestinal diseases or autoimmune disorders. The patient lived with his wife and two children in a suburban area.

On his third visit, the patient presented to the gastroenterology clinic with his wife, who provided additional history. Physical examination revealed pitting edema in both lower extremities, ascites, and mild periorbital edema. Laboratory tests showed severe hypoalbuminemia (serum albumin: 1.8 g/dL), hypoproteinemia (total protein: 4.5 g/dL), and lymphopenia (absolute lymphocyte count: 800 cells/µL). His liver and renal function tests were within normal limits. Stool studies revealed significant fecal alpha-1 antitrypsin loss, indicating PLE.

Given the persistent symptoms and biochemical abnormalities, an upper endoscopy was performed. The endoscopic examination revealed a white villi in the duodenum, suggestive of dilated lacteals. Multiple biopsies were taken from the duodenal mucosa. Histopathological examination confirmed the presence of markedly dilated lymphatic vessels in the lamina propria, consistent with PIL.

Table 1 summarizes the patient's clinical and laboratory data over four visits from April to July 2023 visits. Initially presenting with severe hypoalbuminemia, significant weight loss, and marked edema, the patient's condition showed progressive improvement with treatment. Over the follow-up period, weight increased from 70 kg to 78 kg, serum albumin levels rose from 1.8 g/dL to 3.5 g/dL, and total protein levels improved from 4.5 g/dL to 6.5 g/dL. The edema resolved completely by the third follow-up, and gastrointestinal symptoms subsided. Compliance with the prescribed diet and increasing doses of octreotide contributed to these positive outcomes, reflected in the patient's reported quality of life, which improved from a score of 3 to 9. The diagnosis of PIL was confirmed based on the endoscopic and histopathological findings.

**Table 1:** Patient’s Clinical and Laboratory Findings Over Time

Parameter	Initial Visit (01/04/2023)	1st Follow-Up Visit (02/05/2023)	2nd Follow-Up Visit (04/06/2023)	3rd Follow-Up Visit (04/07/2023)
Weight (kg)	70	72	75	78
Edema (Pitting, Scale 1-4)	3	1	1	0
Serum Albumin (g/dL)	1.8	2.5	3.0	3.5
Total Protein (g/dL)	4.5	5.0	6.0	6.5
Absolute Lymphocyte Count (cells/µL)	800	1000	1200	1400
Fecal Alpha-1 Antitrypsin (mg/g)	120	90	60	30
Gastrointestinal Symptoms (yes/no)	Yes	Yes	No	No
Dietary Compliance (yes/no)	N/A	Yes	Yes	Yes
Octreotide Dosage (mcg, SC TID)	N/A	100	150	200
Patient-Reported Quality of Life (1-10)	3	5	7	9

The patient was managed with a multidisciplinary approach as shown in Table 2. The patient was started on octreotide at a dosage of 100 mg subcutaneously three times daily, which was later adjusted to 200 mg based on clinical response.

**Table 2:** Treatment Regimen and Response MCTs, medium-chain triglycerides

Treatment	Dosage	Duration	Response
High-Protein Diet	1.5 g/kg body weight/day	Continuous	Improved
Low-Fat Diet	<20% of total calories	Continuous	Improved
MCTs	30-40 g/day	Continuous	Improved
Octreotide	100 mcg SC TID initially, increased to 200 mcg SC TID	8 weeks	Significant improvement in symptoms and lab results

Over the following weeks, the patient showed significant clinical improvement. Edema reduced markedly, serum albumin levels increased to 3.5 g/dL, and gastrointestinal symptoms resolved. He was closely monitored with regular follow-up visits and dietary consultations. The patient was advised to continue the dietary modifications and octreotide therapy, with gradual tapering of the dosage as his condition stabilized as shown by the follow-up approach in Table 3.

**Table 3:** Follow-Up and Monitoring

Parameter	Initial Visit	1 Month	3 Months	6 Months	12 Months
Serum Albumin (g/dL)	1.8	2.5	3.0	3.5	4.0
Total Protein (g/dL)	4.5	5.2	6.0	6.5	6.8
Edema (Pitting, Scale 1-4)	3	2	1	0	0
Gastrointestinal Symptoms (Yes/No)	Yes	Yes	No	No	No
Compliance With Diet and Medication	N/A	Yes	Yes	Yes	Yes

**Figure 1:**
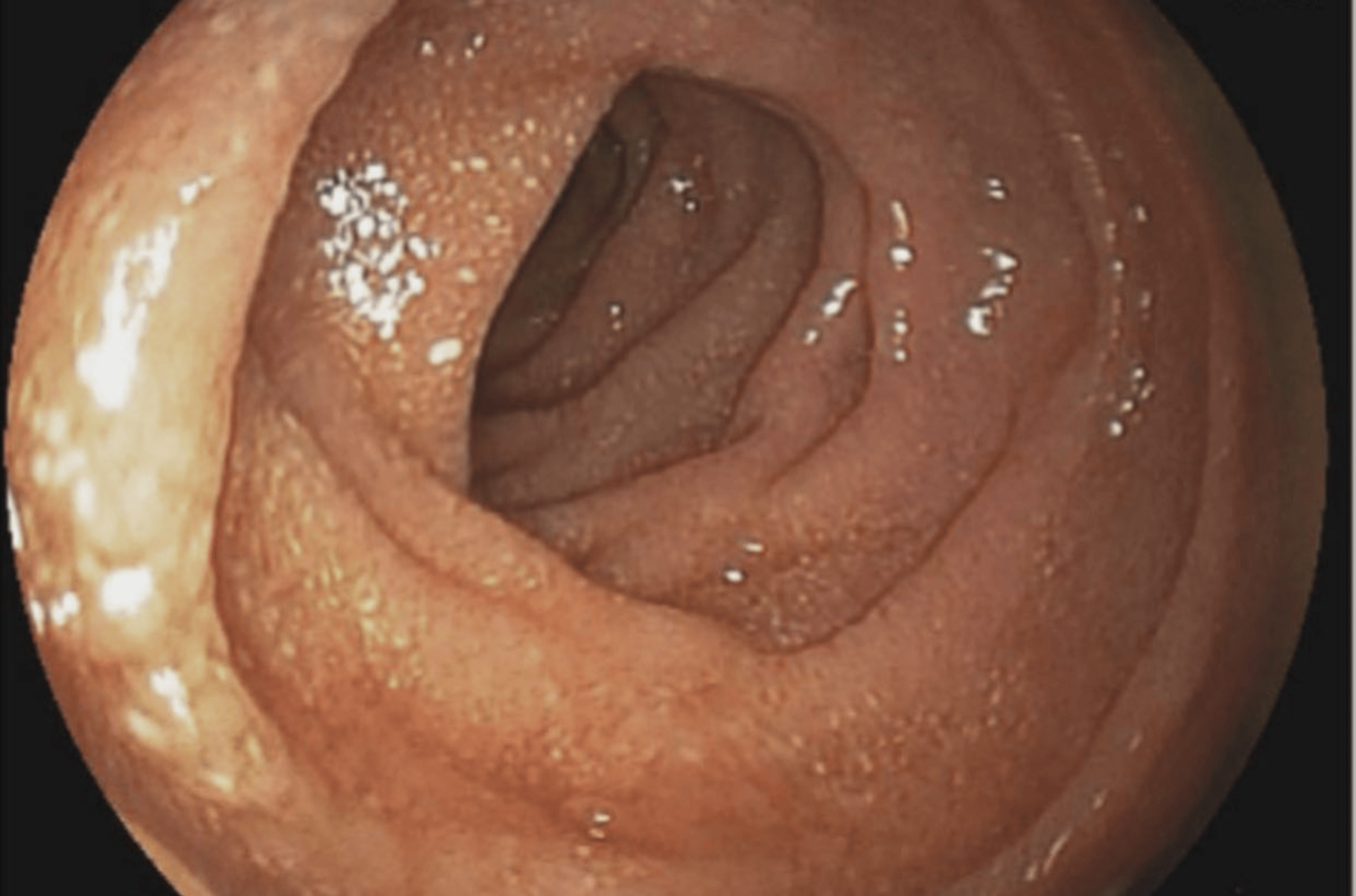
Endoscopic Visualization of Duodenal Mucosa in PIL PIL, primary intestinal lymphangiectasia

Figure 1 shows an endoscopic view of the duodenum. The image depicts the internal mucosal surface of the duodenum. The image shows the mucosal lining of the duodenum but with notable white, patchy areas indicative of dilated lacteals, characteristic of PIL (before treatment). The mucosal surface appears slightly irregular due to these whitish areas. 

**Figure 2:**
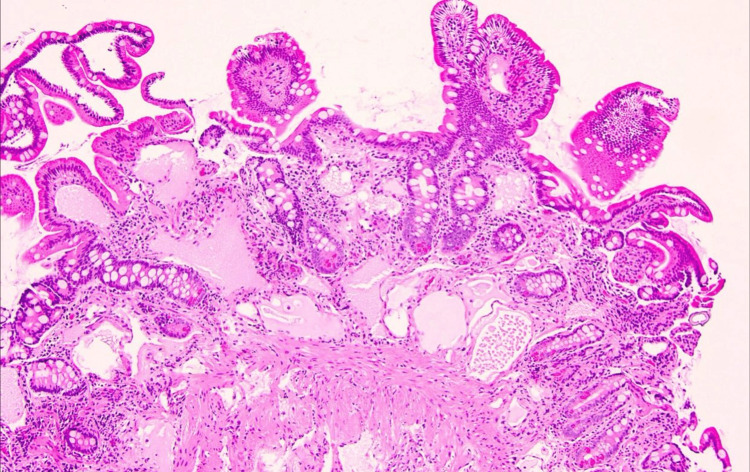
Histopathological Features of Dilated Lacteals in PIL PIL, primary intestinal lymphangiectasia

This histopathological slide in Figure 2 shows a section of the small intestine of the patient stained with hematoxylin and eosin (H&E). The mucosal layer is characterized by finger-like projections called villi, which appear somewhat blunted and distorted, indicative of underlying pathology. The surface of the villi is lined with epithelial cells, primarily enterocytes, responsible for nutrient absorption, and interspersed goblet cells, which secrete mucus to lubricate the intestinal lining. A prominent feature is the presence of markedly dilated lacteals within the villi, which are the lymphatic vessels responsible for transporting absorbed fats from the intestine. The dilation of these lacteals is a key indicator of PIL. The submucosal layer contains loose connective tissue with a network of blood vessels, lymphatics, and immune cells, with evident dilated lymphatic spaces further supporting the diagnosis. There is a moderate infiltration of lymphocytes within the lamina propria, indicating an inflammatory response common in lymphangiectasia due to chronic lymphatic leakage and tissue damage. Additionally, the intestinal crypts, known as the Crypts of Lieberkühn, are present and appear to maintain their structure despite the surrounding changes. 

**Figure 3:**
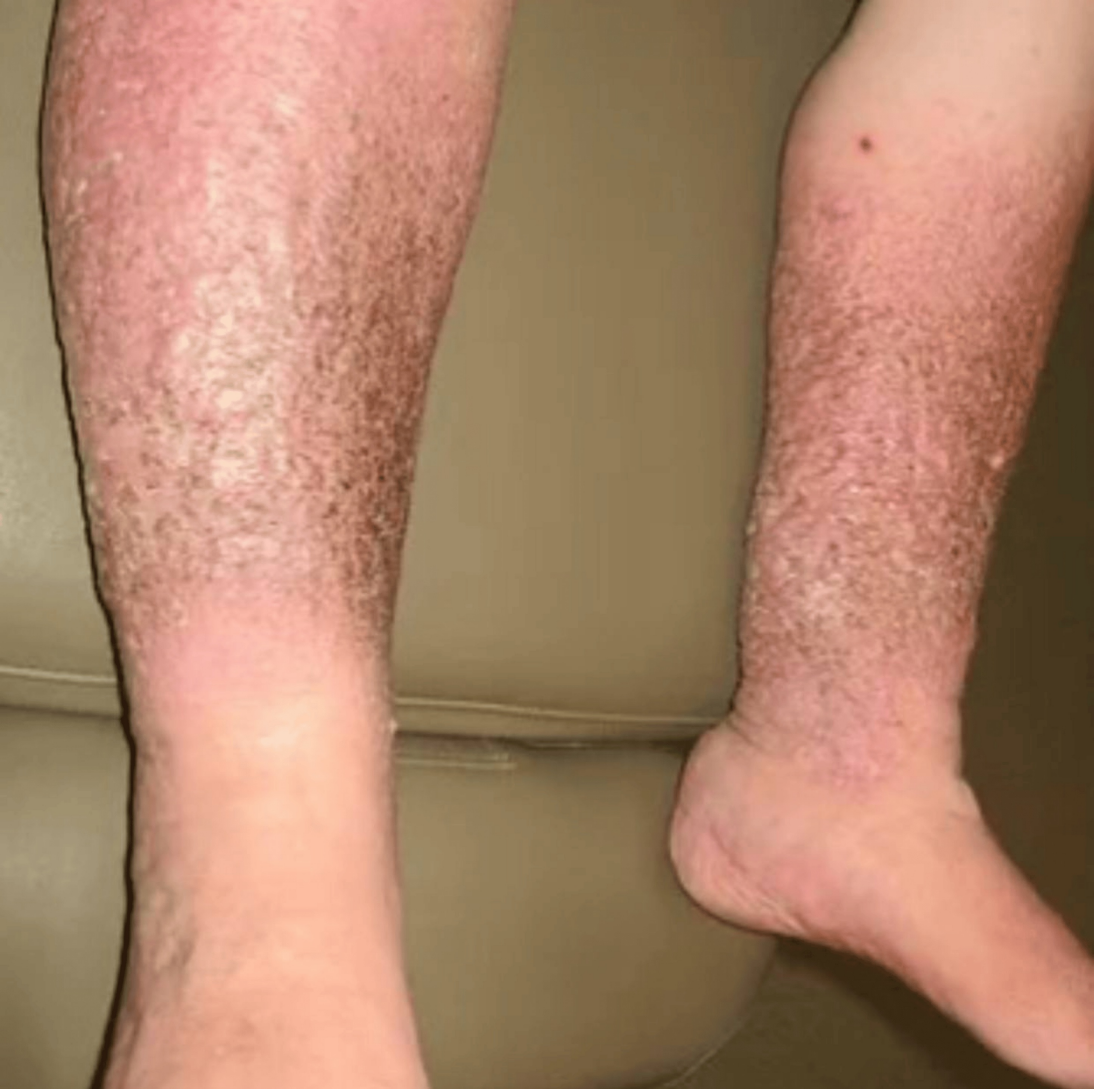
Severe Edema and Hyperpigmentation in PIL PIL, primary intestinal lymphangiectasia

This clinical image shows the lower extremities of our patient who presented with symptoms of refractory PLE as shown in Figure 3. The image was taken to document the patient's physical condition, particularly the severe dermatological manifestations associated with his condition. The skin on the lower legs appears thickened and hyperpigmented, indicative of chronic changes often seen in patients with chronic lymphatic issues and PLE. There are extensive areas of scaling and dryness, which can be a result of severe malnutrition and chronic protein loss. Mild to moderate pitting edema is present, which is a hallmark of hypoalbuminemia and associated PLE, with the edema more pronounced around the ankles and lower calves. The skin shows signs of chronic inflammation, with erythema (redness) and scaling, suggestive of a longstanding inflammatory process. Both legs exhibit similar dermatological changes, suggesting a systemic issue rather than a localized skin condition. 

## Discussion

This case of PIL is unique due to its presentation in an adult patient, which is exceptionally rare, as PIL is typically diagnosed in childhood. The patient's refractory PLE, characterized by significant edema, chronic diarrhea, and severe hypoalbuminemia, persisted despite initial treatments for presumed inflammatory bowel disease. This prolonged and complex clinical course highlights the importance of considering less common etiologies such as PIL [1].

Endoscopy played a critical role in this case by revealing white villi in the duodenum, indicative of dilated lacteals characteristic of PIL. The visual confirmation provided by endoscopy directed the diagnostic process toward a more specific investigation [2]. The histopathological examination of duodenal biopsies was definitive in confirming PIL, demonstrating markedly dilated lymphatic vessels in the lamina propria [4]. Histopathology then provided the microscopic confirmation needed to diagnose PIL. The examination of duodenal biopsy samples revealed the presence of dilated lymphatic vessels in the lamina propria, a hallmark of PIL. This finding was critical in solidifying the diagnosis, as it ruled out other potential causes of the patient's symptoms, such as inflammatory bowel disease, celiac disease, and certain types of gastrointestinal lymphoma.

An important aspect of managing this case involved nutritional support and careful dietary planning, which were crucial in mitigating the symptoms and improving the patient’s quality of life. Given the protein-losing nature of PIL, the patient was placed on a high-protein, low-fat diet supplemented with MCTs, which are easier to absorb and do not rely on the lymphatic system. This dietary adjustment aimed to reduce lymphatic pressure and prevent further dilation of lacteals, thereby decreasing protein loss. The patient’s nutritional status was closely monitored, with regular assessments of serum albumin levels and overall nutritional intake, ensuring that the dietary intervention was effectively addressing hypoalbuminemia and other nutritional deficiencies. The combination of diagnostic tools, endoscopy, and histopathology, was essential in accurately diagnosing PIL in an atypical adult presentation. Each modality provided unique and complementary information that, together, formed a comprehensive understanding of the patient's condition [7-10].

This case report is distinctive in that it documents PIL with PLE in an adult, which is relatively rare since PIL predominantly presents in children. The uniqueness is highlighted by the use of endoscopic and histopathological findings to confirm the diagnosis. The endoscopic imaging revealed characteristic white villi in the duodenum, and histopathology confirmed markedly dilated lymphatic vessels in the lamina propria, which are crucial for diagnosing PIL. Moreover, the patient’s response to treatment with octreotide and dietary modifications provides insight into the management of adult PIL, emphasizing the need for tailored therapeutic approaches. The improvement in clinical parameters, such as weight, serum albumin levels, and gastrointestinal symptoms, alongside the reduction in fecal alpha-1 antitrypsin loss, offers valuable evidence on the efficacy of this treatment regimen in managing adult cases of PIL.

## Conclusions

This case report highlights the rare and atypical presentation of PIL in an adult patient, emphasizing the diagnostic challenges and the necessity for a comprehensive, multimodal approach. The persistence of refractory PLE despite conventional treatments prompted a detailed investigation utilizing endoscopy, CT imaging, and histopathological examination. Each diagnostic modality provided critical insights, collectively leading to the accurate diagnosis of PIL. This case underscores the importance of considering rare etiologies in complex clinical scenarios and demonstrates the value of integrating various diagnostic tools to achieve a definitive diagnosis. The successful management of this patient with dietary modifications and octreotide therapy further illustrates the potential for positive outcomes with targeted treatment strategies.
